# The role of loneliness in smartphone addiction levels of adolescent online game players

**DOI:** 10.3389/fpsyg.2026.1839132

**Published:** 2026-05-12

**Authors:** Mehmet Cuneyt Birkok, Ozkan Isik, Laurentiu-Gabriel Talaghir, Gabriel Marian Manolache

**Affiliations:** 1 Department of Sociology, Faculty of Human and Public Sciences, Sakarya University, Sakarya, Türkiye; 2 Faculty of Sport Sciences, Balikesir University, Balikesir, Türkiye; 3 Faculty of Physical Education and Sport, Dunarea de Jos University of Galati, Galati, Romania

**Keywords:** digital addiction, loneliness, online gaming, smartphone addiction, young adults

## Abstract

**Purpose:**

This study aimed to examine the relationship between loneliness and smartphone addiction among adolescent individuals who engage in online gaming.

**Method:**

The study was designed using a correlational survey model within the framework of quantitative research methods. A total of 405 online game players (211 male and 194 female) voluntarily participated in the study. During the data collection process, the UCLA Loneliness Scale (ULS-8) and the Smartphone Addiction Scale–Short Form (SAS-SF) were utilized. The obtained data were analyzed using SPSS 25.0 software. In this process, the normality of the dataset was examined, descriptive statistics were calculated, and Pearson correlation analysis along with simple linear regression analysis were employed to determine the relationships between variables. All statistical analyses were conducted at a significance level of *p* < 0.05.

**Results:**

The findings of the study indicated a positive and statistically significant relationship between loneliness and smartphone addiction. The results revealed that an increase in the level of loneliness leads to a corresponding increase in smartphone addiction. Furthermore, it was determined that loneliness explains approximately 16.4% of the variance in smartphone addiction.

**Conclusion:**

Loneliness appears to be a significant predictor of problematic digital device use behaviors among adolescents. The study provides important implications for both the evaluation of adolescents’ psychosocial states within online gaming environments and the development of interventions aimed at preventing digital addiction.

## 1 Introduction

In recent years, the rapid advancement of digital technologies has significantly transformed the daily life practices and social interaction patterns of individuals, particularly during adolescence (Martin-Barrado and Gomez-Baya, 2025; Moreno et al., 2021; Sezer and Cengiz, 2025; Er and Cengız, 2025). The period between the ages of 13 and 19 is considered a critical developmental stage in which identity formation, social belonging, and peer relationships are intensively shaped. It has also been emphasized that noticeable declines in face-to-face communication skills occur during this period (Bas et al., 2025), leading adolescents to increasingly turn to digital platforms for establishing social connections, entertainment, and coping with stress (Fan et al., 2025).

Play is an activity that supports learning through enjoyment and contributes to the development of both cognitive and physical skills; however, traditional street games have largely been replaced by digital games played on electronic devices such as smartphones and computers (Demir and Cicioğlu, 2020). In particular, online gaming environments have become a prominent leisure activity by offering both competitive and socially interactive digital spaces for young individuals (Cengiz and Er, 2025; Martin-Barrado and Gomez-Baya, 2025). Although online gaming provides opportunities for social interaction, it does not always guarantee meaningful social connection. In this regard, psychosocial factors such as loneliness may play a crucial role in shaping adolescents’ patterns of digital engagement. Although virtual social networks established through online games may support adolescents’ peer relationships, the psychosocial consequences of intensive digital use have become an important area of interest for researchers. Indeed, recent studies indicate that adolescents’ intensive engagement with digital technologies is closely associated with variables such as loneliness, psychological wellbeing, and problematic technology use (Kabadayi, 2024; Li et al., 2021). Furthermore, it has been reported that increasing online gaming addiction may lead to sedentary lifestyles and negatively affect dietary behaviors, thereby resulting in significant adverse health outcomes (Ayas and Göral, 2023).

In this context, adolescents who engage in online gaming constitute an important research group in terms of problematic digital device use, as they both fulfill their social interaction needs in digital environments and have continuous access to mobile devices. The integration of gaming, social media, and communication tools within smartphones increases screen time among young users and may bring about various psychological and behavioral risks. Within this context, digital gaming emerges as one of the most prominent forms of online engagement among adolescents, combining entertainment, competition, and social interaction in a single environment. Therefore, understanding gaming behaviors is essential for examining adolescents’ digital experiences more comprehensively. Therefore, examining the relationships between online gaming behaviors and psychosocial variables in adolescents is considered crucial for understanding youth mental health in the digital age.

Loneliness is defined as a subjective and negative psychological experience arising from the discrepancy between an individual’s existing social relationships and their desired level of social connection (Hawkley and Cacioppo, 2010). This concept is not merely related to being physically alone but rather to the perceived quality of social relationships and the satisfaction derived from them. In other words, loneliness emerges as an emotional experience when an individual perceives their social bonds as insufficient or unsatisfactory (Nowland et al., 2018). Adolescence is a critical developmental period in which individuals form their social identities and peer relationships gain prominence. Particularly between the ages of 13 and 19, the need for social acceptance and belonging increases significantly. Difficulties in peer relationships or a lack of social support may lead to the emergence of loneliness. Research indicates that loneliness in adolescents is closely associated with psychological wellbeing, social adjustment, and behavioral outcomes (Loades et al., 2020; Shah and Househ, 2023).

With the widespread use of digital technologies, the relationship between loneliness and online behaviors has also attracted increasing scholarly attention. Today, adolescents conduct a significant portion of their social interactions in online environments. Social media platforms and online games provide alternative means of establishing social connections. However, some studies suggest that individuals who primarily meet their social needs in digital environments may experience higher levels of loneliness (Boursier et al., 2020; Zhao et al., 2024). Various theoretical approaches have been developed in the literature to explain the relationship between loneliness and digital technology use. One of these, the Compensatory Internet Use Theory, posits that individuals turn to the internet and digital environments to compensate for unmet social or emotional needs in real life. According to this theory, individuals experiencing loneliness tend to spend more time in online environments to achieve social interaction and psychological relief (Kardefelt-Winther, 2014). Similarly, the Uses and Gratifications Theory explains that individuals use media to satisfy psychological needs such as entertainment, social interaction, and escape. Within this framework, it is suggested that individuals experiencing loneliness are more likely to turn to digital platforms and online games to fulfill their social needs (Bányai et al., 2021).

Recent empirical studies also reveal significant relationships between loneliness and problematic digital device use behaviors (Du et al., 2023; Meckovsky et al., 2023). Research conducted on adolescents shows that those with higher levels of loneliness tend to use smartphones more intensively and are more prone to problematic usage behaviors (Li et al., 2021; Zhao et al., 2024). These findings indicate that loneliness is a key psychological factor associated with excessive digital technology use. In this regard, loneliness is considered a critical variable influencing adolescents’ digital technology usage behaviors. Indeed, it has been noted that the dynamics between adolescent loneliness and problematic smartphone use have not been sufficiently examined (Zhao et al., 2024). At this point, smartphones—being central to individuals’ daily lives—offer a significant research context for understanding the relationship between loneliness and problematic digital device use.

Smartphone addiction is defined as a problematic digital behavior characterized by an individual’s inability to control device use, increased duration of use, and negative impacts on academic, social, or psychological functioning (Busch and McCarthy, 2021; Sohn et al., 2019). This condition is considered within the scope of non-substance behavioral addictions and may significantly affect quality of life and psychological wellbeing, particularly among adolescents (Kwon et al., 2013; Panova and Carbonell, 2018). Studies indicate that smartphone use among adolescents has increased due to the integration of multiple functions such as internet access, social media, instant messaging, and mobile gaming. This intensive usage is associated with sleep disturbances, academic failure, social isolation, and psychological distress (Li et al., 2021; Sohn et al., 2019).

Various theoretical models have been proposed to explain smartphone addiction. The I-PACE Model (Brand et al., 2016) suggests that problematic internet and technology use is shaped by the interaction of individual characteristics, emotional states, cognitive processes, and executive control mechanisms. According to this model, psychological conditions such as loneliness, social anxiety, and depression may increase problematic smartphone use. The Cognitive Behavioral Model of Problematic Internet Use similarly posits that negative cognitive and psychological processes may lead to excessive digital technology use (Tokunaga, 2014). Likewise, the Compensatory Internet Use Theory argues that individuals turn to digital platforms to fulfill social and emotional needs, with loneliness being a key triggering factor (Kardefelt-Winther, 2014).

Empirical studies also support the relationship between loneliness and smartphone addiction. Research on adolescents indicates that individuals with higher levels of loneliness tend to use smartphones more intensively and are more susceptible to problematic usage behaviors (Li et al., 2021; Zhao et al., 2024; Elhai et al., 2020). In particular, among adolescents who engage in online gaming, the use of devices for both gaming and social communication increases the risk of problematic use (Bányai et al., 2021; Sohn et al., 2019). In this context, examining the impact of psychosocial variables such as loneliness on smartphone addiction is of critical importance for understanding digital behaviors among adolescent online gamers.

Despite the growing body of research examining the relationship between loneliness and smartphone addiction, several important gaps remain in the literature. First, previous studies have largely focused on general adolescent populations, while relatively limited attention has been given to adolescents who actively engage in online gaming. This group represents a unique population because they use smartphones simultaneously for gaming, social interaction, and communication. Second, although the association between loneliness and problematic smartphone use has been documented, the specific mechanisms underlying this relationship within online gaming contexts remain underexplored. In particular, integrating gaming and social interaction in digital environments may intensify the impact of psychosocial factors, such as loneliness, on smartphone use behaviors. Therefore, this study aims to address these gaps by focusing specifically on adolescent online game players and examining the predictive role of loneliness in smartphone addiction within this context.

The primary aim of this study was to examine the relationship between loneliness and smartphone addiction among adolescents aged 13–19 who engage in online gaming. In doing so, the study provides a more context-specific understanding of problematic smartphone use by focusing on a group characterized by intensive and multifunctional digital engagement. Additionally, the study aims to examine the predictive role of loneliness in smartphone addiction and to identify the psychosocial determinants of smartphone use among adolescent online gamers. In this way, the study contributes to the existing literature by offering insights into how loneliness operates as a risk factor within digitally embedded social environments.

In line with this aim, the hypotheses of the study are as follows:

*H*_1_: There is a positive relationship between loneliness levels and smartphone addiction among adolescents who engage in online gaming.

*H*_2_: Loneliness significantly predicts smartphone addiction.

## 2 Materials and methods

### 2.1 Research design

This study aims to examine the effect of loneliness on smartphone addiction among adolescents who engage in online gaming. The research was designed within the framework of a quantitative approach, employing a correlational survey model to identify the relationships among variables. The correlational survey model enables the examination of associations between variables and allows for the evaluation of potential predictive relationships based on these associations. In this context, the interaction between loneliness and smartphone addiction was analyzed in a systematic manner (Christensen et al., 2020; Karasar, 2024).

### 2.2 Participants

A convenience sampling method was employed to determine the study group. This approach allows researchers to include individuals who are readily accessible in the sample and facilitates data collection from participants who are easy to reach until the targeted sample size is achieved (Cohen et al., 2011). To determine the required sample size, a power analysis was conducted using the G*Power statistical software. The results indicated that, with an 80% statistical power (1-β), a 5% significance level (α), and an effect size of 0.20, the minimum required sample size for correlation analysis was 193 participants (Richard et al., 2003).

Considering potential data loss and participant attrition, it was deemed appropriate to exceed this minimum threshold. Accordingly, a total of 405 adolescents who reported playing at least one online game and voluntarily agreed to participate were included in the study. Of the participants, 47.9% were female (n = 194; age range: 14–19; mean age = 16.65 ± 0.99), and 52.1% were male (n = 211; age range: 13–19; mean age = 16.42 ± 1.20). Although the use of a convenience sampling method may limit the generalizability of the findings to the broader adolescent population, the relatively large sample size and the diversity within the online gaming group help to enhance the robustness and external validity of the results within similar contexts.

### 2.3 Data collection tools

Data were collected using a Personal Information Form (age and gender), the UCLA Loneliness Scale, and the Smartphone Addiction Scale–Short Form.

#### 2.3.1 UCLA Loneliness Scale (ULS-8)

Originally developed by Hays and DiMatteo (1987) and adapted into Turkish by Yıldız and Duy (2014), this scale has a unidimensional structure and consists of 7 items in its Turkish version. Although the original form included 8 items, one item was removed during the adaptation process due to insufficient factor loading (>0.30). The items are rated on a four-point Likert scale, and item 5 is reverse-coded. Total scores range from 7 to 28, with higher scores indicating greater levels of loneliness. Reliability analyses conducted during the Turkish adaptation reported a Cronbach’s alpha internal consistency coefficient of 0.74 and a test–retest reliability coefficient of 0.84. Confirmatory factor analysis (CFA) results demonstrated acceptable model fit (CMIN/DF = 1.094, GFI = 0.97, CFI = 0.98, RMSEA = 0.06, SRMR = 0.04). Additionally, the composite reliability (CR) value was calculated as 0.75, and the average variance extracted (AVE) value as 0.40.

#### 2.3.2 Smartphone Addiction Scale–Short Form (SAS-SF)

Developed by Kwon et al. (2013) and adapted into Turkish by Noyan et al. (2015), this scale consists of 10 items and has a single-factor structure. It is rated on a six-point Likert scale, and no reverse-coded items are included. Total scores range from 10 to 60, with higher scores indicating higher levels of smartphone addiction. In the adaptation study, the Cronbach’s alpha coefficient was reported as 0.867, and the test–retest reliability coefficient as 0.926, indicating a high level of reliability.

#### 2.3.3 Validity and reliability of measurement tools

To assess the construct validity of the measurement instruments used in this study, first-order Confirmatory Factor Analysis (CFA) was conducted separately for each scale. Given that both scales have a unidimensional structure, analyses were performed using a first-order factor model. Since the data met the assumption of multivariate normality, the Maximum Likelihood estimation method was employed. CFA results were evaluated based on commonly accepted goodness-of-fit indices in the literature, and theoretically justified modifications were applied where necessary to improve model fit. In addition, Cronbach’s alpha coefficients were calculated to assess the internal consistency of the scales, and the results were presented in tabular form. The evaluation of model fit was based on the criteria recommended by Byrne (2016) and Gürbüz (2021). For interpreting reliability levels, the guidelines proposed by George and Mallery (2019) were followed, whereby Cronbach’s alpha values above 0.80 indicate good reliability and values above 0.90 indicate excellent reliability. Furthermore, Average Variance Extracted (AVE) and Composite Reliability (CR) values were examined to assess construct validity. According to Fornell and Larcker (1981), AVE values above 0.50 and CR values above 0.70 indicate adequate validity. However, Maruf et al. (2021) suggest that an AVE value above 0.40 may also be considered acceptable if the CR value is within an acceptable range. Accordingly, the CFA results, skewness and kurtosis values, Cronbach’s alpha coefficients, as well as AVE and CR values for the measurement instruments are presented in Table 1.

**TABLE 1:** CFA fit indices, skewness–kurtosis, Cronbach’s alpha, AVE, and CR values of the measurement instruments.

Index	Good fit	Acceptable fit	UCLA loneliness	SAS-SF
X^2^/df	<3	<3(X^2^/df) < 5	1.502	3.177
GFI	>0.95	>0.90	0.987	0.952
CFI	>0.95	>0.90	0.994	0.957
RMSEA	<0.05	<0.08	0.035	0.073
SRMR	<0.05	<0.08	0.217	0.043
Cronbach’s alpha	>0.90	>0.80	0.806	0.881
Skewness	–	-1/+1	0.703	0.404
Kurtosis	–	-1/+1	0.135	-0.412
AVE	>0.50	>0.40	0.449	0.417
CR	>0.70	–	0.825	0.876

GFI, Goodness-of-Fit Index; CFI, Comparative Fit Index; RMSEA, Root Mean Square Error of Approximation; SRMR, Standardized Root Mean Square Residual; AVE, Average Variance Extracted; CR, Composite Reliability.

### 2.4 Ethical approval

This research was approved by the Non-Interventional Research Ethics Committee of Balıkesir University Faculty of Health Sciences under protocol number 2026/74. Following the approval process, the parents or legal guardians of the adolescents who were planned to participate in the study were informed about the research. Since the participants were under the age of 18, written informed parental consent was obtained from their legal guardians. Subsequently, informed consent was also obtained from the adolescent participants themselves, confirming their voluntary participation and their agreement that the collected data would be used solely for scientific purposes. Participants were clearly informed of their right to withdraw from the study at any stage of the research process without any consequences. All procedures carried out within the scope of the study were conducted in accordance with the ethical principles outlined in the Declaration of Helsinki (1964) and its subsequent revisions. The data collection process was conducted through online survey forms and consent documents obtained from voluntary participants who engage in online gaming.

### 2.5 Statistical analysis

Prior to the statistical analyses, the dataset was examined using the SPSS 25.0 statistical software package. In this stage, the dataset was screened for outliers and missing data. As a result of this examination, data from 23 participants were identified as either incomplete or systematically filled out and were therefore excluded from the dataset. Consequently, all statistical analyses were conducted based on the data obtained from 405 adolescents. The normality of the data distribution was assessed by examining skewness and kurtosis coefficients. Values within the range of ± 1 indicate that the data can be considered normally distributed and that parametric tests can be appropriately applied (Hair et al., 2010). In addition to descriptive statistics, Pearson correlation analysis was performed to determine the relationships between variables, and simple linear regression analysis was conducted to examine predictive effects. The level of statistical significance was set at 0.05 for all analyses. Furthermore, descriptive statistics for the scale scores, including mean, standard deviation, minimum, and maximum values, are presented in Table 2.

**TABLE 2:** Descriptive statistics of scale scores.

Scale	n	Min-max	X̄ ± SD
UCLA loneliness	405	7–28	12.78 ± 4.67
Smartphone addiction (SAS-SF)	405	10–60	28.27 ± 10.81

## 3 Results

The findings revealed a positive, statistically significant, and moderate relationship between loneliness and smartphone addiction among adolescents who engage in online gaming (*r* = .0404, *p* < 0.05). This result indicates that as loneliness levels increase, smartphone addiction levels also tend to increase (Table 3).

**TABLE 3:** Relationship between loneliness and smartphone addiction.

Variables		Smartphone addiction (SAS-SF)
UCLA loneliness	*r*	0.404
	*P*	0.001*

*p < 0.05.

**FIGURE 1:**
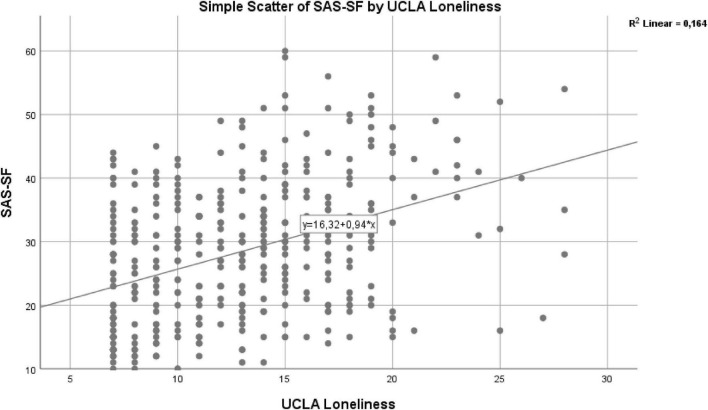
Scatter plot showing the relationship between loneliness and smartphone addiction with regression line.

The regression analysis demonstrated that loneliness is a statistically significant predictor of smartphone addiction (*R*^2^ = 0.164, *p* < 0.05). Specifically, loneliness accounts for approximately 16.4% of the variance in smartphone addiction. An examination of the standardized coefficient (β = 0.404) and *t*-value indicates that an increase in loneliness is associated with higher levels of smartphone addiction (Table 4). The scatter plot illustrating this relationship is presented in Figure 1.

**TABLE 4:** Regression Analysis Results for the Predictive Role of Loneliness on Smartphone Addiction.

Variable	*B*	SE	β	*t*	*p*
Constant	16.315	1.434	–	11.374	0.001*
UCLA loneliness	0.936	0.105	0.404	8.875	0.001*
*R* = 0.404; *R*^2^ = 0.164; *F* = 78.772; *p* < 0.05					

*p < 0.05.

## 4 Discussion

This study aimed to systematically and comprehensively investigate the relationship between loneliness and smartphone addiction among adolescents aged 13–19 who engage in online gaming. The findings demonstrated that increases in loneliness are associated with a notable rise in problematic smartphone use behaviors. The regression analysis further indicated that loneliness accounts for approximately 16.4% of the variance in smartphone addiction. This indicates a small-to-moderate effect size, suggesting that loneliness is an important but not sole determinant of smartphone addiction. This finding strongly suggests that loneliness functions as a significant psychosocial determinant in the emergence and development of digital addiction behaviors among adolescents. The results also highlight the sensitivity of adolescence as a developmental period characterized by heightened social, emotional, and psychological vulnerability. In this context, experiences of loneliness may drive adolescents to seek social connection through digital platforms, thereby increasing the likelihood of problematic engagement with digital devices. The findings of the present study are highly consistent with existing literature. Previous research has consistently reported a positive association between loneliness and smartphone addiction (Li et al., 2021; Yalçın et al., 2020; Zhao et al., 2024). Moreover, studies conducted by Bányai et al. (2021), Marino et al. (2018), and Demirci et al. (2015) demonstrate that loneliness plays a crucial role in shaping problematic digital device use, particularly among adolescents involved in online gaming. In addition, findings reported by Boursier et al. (2020) and Ebesutani et al. (2015) indicate that social isolation intensifies feelings of loneliness and increases individuals’ tendency to engage with virtual communities. Furthermore, smartphone addiction has been shown to be significantly associated with broader psychosocial outcomes, such as life satisfaction, underscoring its wider impact on individual wellbeing (Kula et al., 2020).

Similarly, Hunt et al. (2018) and Musetti et al. (2020) emphasize that loneliness functions as a risk factor for the development of problematic internet use behaviors. Furthermore, the findings of Bozoglan et al. (2013) and Nowland et al. (2018) suggest the existence of a reciprocal and cyclical relationship between loneliness and excessive internet use, indicating that this relationship is not unidirectional; rather, problematic use may also exacerbate loneliness over time. Supporting this perspective, Hamaker et al. (2015) and MacDonald and Schermer (2021) report that adolescents with higher levels of loneliness are more prone to persistent and problematic smartphone use, reinforcing the predictive role of loneliness in digital addiction behaviors.

From a theoretical standpoint, the findings can be interpreted within the framework of the I-PACE model, which posits that psychological states, cognitive processes, and emotional needs interact to influence excessive and uncontrolled digital behaviors (Brand et al., 2016). Within this model, loneliness emerges as a critical psychosocial trigger that increases vulnerability to problematic technology use. Similarly, the Compensatory Internet Use Theory suggests that adolescents may attempt to compensate for unmet social and emotional needs—such as loneliness and social isolation—through increased engagement with digital platforms, including smartphones and online gaming environments (Kardefelt-Winther, 2014). In parallel, the Cognitive Behavioral Model of Problematic Internet Use highlights the role of maladaptive cognitive and emotional processes in driving excessive digital device use, thereby strengthening the link between loneliness and problematic usage patterns (Tokunaga, 2014; Zhou and Feng, 2025). Importantly, evidence from the literature emphasizing reciprocal interactions suggests that the relationship between loneliness and problematic use should not be conceptualized as strictly unidirectional. Instead, it is more accurately understood as a bidirectional and self-reinforcing cycle, which necessitates caution when interpreting causal relationships between loneliness and smartphone addiction.

The findings also underscore that adolescents’ need for social connection and experiences of loneliness operate as key mechanisms shaping digital behaviors. Adolescence is widely recognized as a developmental stage marked by significant changes in identity formation, social competence, and emotional regulation (Jimenez et al., 2023). In this context, online games should not be viewed solely as leisure activities; rather, they serve as social environments where adolescents establish connections, experience belonging, and develop interpersonal skills. Rehbein et al. (2021) further indicate that increased engagement in online gaming may be associated with loneliness and social isolation, while simultaneously contributing to higher levels of smartphone-related problematic behaviors. This study offers meaningful contributions to both theoretical understanding and practical application. From a theoretical perspective, the findings support models such as the I-PACE framework and the Compensatory Internet Use Theory, which explain how unmet social and emotional needs may lead to increased digital engagement and, over time, problematic use.

From a practical standpoint, the results highlight the importance of early identification of loneliness in adolescents and the development of targeted interventions. Supporting adolescents’ social and emotional needs in offline environments—particularly through family and educational contexts—may help mitigate the risk of digital addiction. Additionally, designers of digital platforms may consider implementing features that promote healthier and more balanced usage patterns. Furthermore, previous research indicates that students who engage in regular physical activity tend to exhibit lower levels of internet addiction (Karadağ et al., 2025), while individuals with higher levels of smartphone addiction may display less favorable attitudes toward physical activity (Altinisik et al., 2025). These findings suggest that encouraging participation in sports and physical activities may represent an effective strategy for reducing smartphone addiction among adolescents.

In conclusion, this study provides a detailed examination of the interaction between loneliness and smartphone addiction among adolescents engaged in online gaming, integrating both empirical findings and theoretical perspectives. The results demonstrate that loneliness functions as both a predictor and an amplifying factor of problematic digital use, highlighting the critical role of psychosocial variables in understanding digital behaviors. However, the reciprocal nature of this relationship and potential age-related variations should be carefully considered when interpreting the findings and their generalizability.

### 4.1 Strengths, limitations, and future directions

#### 4.1.1 Strengths

One of the primary strengths of this study lies in its relatively large and adequately powered sample consisting of adolescents aged 13–19. With a total of 405 participants, the study provides sufficient statistical power to examine the relationship between loneliness and smartphone addiction. Additionally, the use of well-established measurement instruments—the UCLA Loneliness Scale and the Smartphone Addiction Scale–Short Form—enhances the methodological rigor of the study. The application of confirmatory factor analysis (CFA) within the current sample further strengthens the validity and reliability of the findings. Another notable strength is the examination of the relationship between loneliness and smartphone addiction specifically within the context of online gaming. Given adolescents’ increasing engagement with digital environments, this focus offers a contextually relevant and original contribution to the literature. Finally, linking the findings to established theoretical frameworks (I-PACE, Compensatory Internet Use Theory, and Cognitive Behavioral models) further enhances the theoretical contribution of the study.

#### 4.1.2 Limitations

Despite its contributions, the study has several limitations. First, the use of a convenience sampling method may limit the generalizability of the findings. Since the sample consists exclusively of voluntary adolescents who engage in online gaming, comparisons with other adolescent populations are not possible. Second, the reliance on self-report measures introduces the possibility of response bias, as participants’ perceptions and honesty may influence the accuracy of the data. Finally, the cross-sectional design of the study precludes causal inferences regarding the relationship between loneliness and smartphone addiction.

#### 4.1.3 Future directions

Future studies may employ longitudinal designs to better capture changes in loneliness and the development of smartphone addiction over time. Such approaches would provide stronger evidence regarding causal relationships. Additionally, including diverse demographic groups and cultural contexts would enhance the generalizability of findings. For example, comparative studies involving adolescents from different socioeconomic backgrounds or those who do not engage in online gaming could provide broader insights into the relationship between loneliness and digital addiction. Incorporating qualitative methods or mixed-methods designs may also offer a deeper understanding of adolescents’ lived experiences of loneliness and their motivations for digital engagement. Finally, future research may explore the effectiveness of psychosocial support and intervention programs targeting loneliness and problematic smartphone use, thereby contributing to both theoretical and practical advancements.

## 5 Conclusion

This study revealed a significant and moderate positive relationship between loneliness and smartphone addiction among adolescents aged 13–19 who engage in online gaming. The findings suggest that loneliness may act as a driving force behind excessive and uncontrolled digital behaviors. From a theoretical perspective, the results are consistent with the I-PACE model, Compensatory Internet Use Theory, and cognitive-behavioral frameworks, all of which emphasize the role of psychosocial deficits in shaping problematic technology use. Loneliness, as a psychosocial gap, appears to increase adolescents’ tendency to seek social connection and gratification in digital environments, thereby elevating the risk of smartphone addiction. Within the context of online gaming, these environments may simultaneously serve as mechanisms for alleviating loneliness while also reinforcing problematic usage patterns. Overall, the study contributes both to the academic literature and to practical efforts aimed at understanding and preventing problematic digital behaviors among adolescents. The findings clearly underscore the importance of addressing loneliness as a key factor in promoting healthier digital usage habits and supporting adolescent wellbeing.

## Data Availability

The raw data supporting the conclusions of this article will be made available by the authors, without undue reservation.

## References

[B1] Altinisik U. Guler H. Isik O. Talaghir L. G. Nanu L. Ivan P. (2025). Reflections of digital slavery in physical inactivity: Examining gender-based physical activity attitudes and smartphone addiction among university students. *Front. Public Health* 13:1742639. 10.3389/fpubh.2025.1742639 41561845 PMC12812915

[B2] Ayas M. Göral K. (2023). Investigation of the relationships between digital game addiction, nutritional attitudes and body mass ındex values of 12-14 year old children. *Eur. J. Sport Sci. Educ.* 5 1–10. 10.47778/ejsse.1199423

[B3] Bányai F. Zsila Á Kökönyei G. Griffiths M. D. Demetrovics Z. Király O. (2021). The moderating role of coping mechanisms and being an e-sport player between psychiatric symptoms and gaming disorder: Online survey. *JMIR Mental Health* 8:e21115. 10.2196/21115 33755024 PMC8077919

[B4] Bas M. Tanoglu G. Aksu A. (2025). Spor eğitimi alan üniversite öğrencilerinde teknoloji bağımlılığı ve spora yönelik tutumun incelenmesi. *Sportif Bakış: Spor Eğitim Bilimleri Dergisi* 12 356–373. Turkish. 10.70736/spjses.325

[B5] Boursier V. Gioia F. Musetti A. Schimmenti A. (2020). Facing loneliness and anxiety during the COVID-19 isolation: The role of excessive social media use in a sample of Italian adults. *Front. Psychiatry* 11:586222. 10.3389/fpsyt.2020.586222 33363484 PMC7752864

[B6] Bozoglan B. Demirer V. Sahin I. (2013). Loneliness, self-esteem, and life satisfaction as predictors of Internet addiction: A cross-sectional study among Turkish university students. *Scand. J. Psychol.* 54 313–319. 10.1111/sjop.12049 23577670

[B7] Brand M. Young K. S. Laier C. Wölfling K. Potenza M. N. (2016). Integrating psychological and neurobiological considerations regarding the development and maintenance of specific Internet-use disorders: An Interaction of Person-Affect-Cognition-Execution (I-PACE) model. *Neurosci. Biobehav. Rev.* 71 252–266. 10.1016/j.neubiorev.2016.08.033 27590829

[B8] Busch P. A. McCarthy S. (2021). Antecedents and consequences of problematic smartphone use: A systematic literature review of an emerging research area. *Comp. Hum. Behav.or* 114:106414. 10.1016/j.chb.2020.106414

[B9] Byrne B. M. (2016). *Structural Equation Modeling with AMOS.* New York, NY: Routledge.

[B10] Cengiz R. Er B. (2025). Digital gaming participation from a serious leisure perspective examining digital literacy and mindful attention awareness levels. *Sportif Bakış: Spor Eğitim Bilimleri Dergisi* 12 148–160. 10.70736/spjses.292

[B11] Christensen L. B. Johnson R. B. Turner L. A. (2020). *Araştırma yöntemleri: Desen ve analiz.* Ankara: Anı Yayıncılık. Turkish

[B12] Cohen S. Manion L. Morrison K. (2011). *Research Methods in Education*, 7th Edn. Routledge.

[B13] Demir G. T. Cicioğlu H. I (2020). Awareness of digital game addiction scale (ADGAS): Validity and reliability study. *Eur. J. Sport Sci. Educ.* 2 1–17. 10.47778/ejsse.5

[B14] Demirci K. Akgönül M. Akpinar A. (2015). Relationship of smartphone use severity with sleep quality, depression, and anxiety in university students. *J. Behav. Addict.* 4 85–92. 10.1556/2006.4.2015.010 26132913 PMC4500888

[B15] Du X. Xiang G. Xiao M. Liu X. Sun J. Ding C. et al. (2023). The relationship between loneliness and problematic smartphone use among adolescents during the COVID-19 pandemic: The mediating role of negative emotions and maladaptive coping. *J. Adolesc.* 95 1449–1462. 10.1002/jad.12218 37435881

[B16] Ebesutani C. Fierstein M. Viana A. G. Trent L. Young J. Sprung M. (2015). The role of loneliness in the relationship between anxiety and depression in clinical and school-based youth. *Psychol. Schools* 52 223–234. 10.1002/pits.21818

[B17] Elhai J. D. Yang H. Fang J. Bai X. Hall B. J. (2020). Depression and anxiety symptoms are related to problematic smartphone use severity in Chinese young adults: Fear of missing out as a mediator. *Add. Behav.* 101:105962. 10.1016/j.addbeh.2019.04.020 31030950

[B18] Er B. Cengız R. (2025). The form of happiness in the digital age: Examining the effect of ınternet usage in digital leisure on flow experience. *Intern. J. Recreat. Sports Sci.* 9 29–44. 10.46463/ijrss.1608338

[B19] Fan T. Bliss L. Calvin A. Selkie E. (2025). Adolescents’ social media posting, social support, and the moderating role of tech attitudes and self-esteem: A 2-year longitudinal study. *Front. Psychol.* 16:1561581. 10.3389/fpsyg.2025.1561581 40900946 PMC12399651

[B20] Fornell C. Larcker D. F. (1981). Evaluating structural equation models with unobservable variables and measurement error. *J. Market. Res.* 18 39–50. 10.1177/002224378101800104

[B21] George D. Mallery P. (2019). *IBM SPSS Statistics 26 Step by Step: A Simple Guide and Reference.* New York, NY and London: Routledge.

[B22] Gürbüz S. (2021). *Aracı ve düzenleyici etki analizleri.* Ankara: Seçkin Yayıncılık. Turkish

[B23] Hair J. F. Black W. C. Babin B. J. Anderson R. E. (2010). *Multivariate Data Analysis*, 7th Edn. New York, NY: Pearson.

[B24] Hamaker E. L. Kuiper R. M. Grasman R. P. P. P. (2015). A critique of the cross-lagged panel model. *Psychol. Methods* 20 102–116. 10.1037/a0038889 25822208

[B25] Hawkley L. C. Cacioppo J. T. (2010). Loneliness matters: A theoretical and empirical review of consequences and mechanisms. *Ann. Behav. Med.* 40 218–227. 10.1007/s12160-010-9210-8 20652462 PMC3874845

[B26] Hays R. D. DiMatteo M. R. (1987). A short-form measure of loneliness. *J. Personal. Assess.* 51 69–81. 10.1207/s15327752jpa5101_6 3572711

[B27] Hunt M. G. Marx R. Lipson C. Young J. (2018). No more FOMO: Limiting social media decreases loneliness and depression. *J. Soc. Clin. Psychol.* 37 751–768. 10.1521/jscp.2018.37.10.751

[B28] Jimenez A. L. Banaag C. G. Arcenas A. M. A. Hugo L. V. (2023). “Adolescent development,” in *Tasman’s Psychiatry*, eds Tasman A. Riba M. B. Alarcón R. D. Alfonso C. A. Kanba S. Lecic-Tosevski D. et al. (Cham: Springer), 1–43. 10.1007/978-3-030-42825-9_106-1

[B29] Kabadayi F. (2024). Smartphone addiction, depression, distress, eustress, loneliness, and sleep deprivation in adolescents: A latent profile and network analysis approach. *BMC Psychol.* 12:608. 10.1186/s40359-024-02117-6 39478568 PMC11526649

[B30] Karadağ Ö Çoknaz H. Memedoğlu I Keser S. (2025). Examining of high school students’ internet addiction levels in terms of sports participation and various variables. *Eur. J. Sport Sci. Educ.* 7 204–215. 10.47778/ejsse.1698309

[B31] Karasar N. (2024). *Bilimsel araştırma yöntemleri (39. baskı).* Ankara: Nobel Yayıncılık. Turkish

[B32] Kardefelt-Winther D. (2014). A conceptual and methodological critique of internet addiction research: Towards a model of compensatory internet use. *Comp. Hum. Behav.* 31 351–354. 10.1016/j.chb.2013.10.059

[B33] Kula H. Ayhan C. Soyer F. Kaçay Z. (2020). The relationship between smartphone addiction and life satisfaction: Faculty of sport sciences students. *Intern. J. Psychol. Educ. Stud.* 7 86–95. 10.17220/ijpes.2020.01.008

[B34] Kwon M. Kim D. J. Cho H. Yang S. (2013). The smartphone addiction scale: Development and validation of a short version for adolescents. *PLoS One* 8:e83558. 10.1371/journal.pone.0083558 24391787 PMC3877074

[B35] Li J. Zhan D. Zhou Y. Gao X. (2021). Loneliness and problematic mobile phone use among adolescents during the COVID-19 pandemic: The roles of escape motivation and self-control. *Add. Behav.* 118:106857. 10.1016/j.addbeh.2021.106857 33676160 PMC8598166

[B36] Loades M. E. Chatburn E. Higson-Sweeney N. Reynolds S. Shafran R. Brigden A. et al. (2020). Rapid systematic review: The impact of social isolation and loneliness on the mental health of children and adolescents in the context of COVID-19. *J. Am. Acad. Child Adolesc. Psychiatry* 59 1218–1239. 10.1016/j.jaac.2020.05.009 32504808 PMC7267797

[B37] MacDonald K. B. Schermer J. A. (2021). Loneliness unlocked: Associations with smartphone use and personality. *Acta Psychol.* 221:103454. 10.1016/j.actpsy.2021.103454 34844066

[B38] Marino C. Gini G. Vieno A. Spada M. M. (2018). A comprehensive meta-analysis on problematic Facebook use. *Comp. Hum. Behav.* 83 262–277. 10.1016/j.chb.2018.02.009

[B39] Martin-Barrado A. D. Gomez-Baya D. (2025). The association between the use of digital technologies and positive youth development: A systematic review. *Front. Psychol.* 16:1552128. 10.3389/fpsyg.2025.1552128 40688549 PMC12272609

[B40] Maruf T. I. Manaf N. H. B. A. Haque A. K. M. A. Maulan S. B. (2021). Factors affecting attitudes towards using ride-sharing apps. *Intern. J. Bus. Econ. Law* 25 60–70.

[B41] Meckovsky F. Furstova J. Kosarkova A. Meier Z. Tavel P. Malinakova K. (2023). Loneliness is associated with problematic internet use but not with the frequency of substance use: A Czech cross-sectional study. *Intern. J. Public Health* 68:1606537. 10.3389/ijph.2023.1606537 38024207 PMC10651728

[B42] Moreno M. A. Binger K. Zhao Q. Eickhoff J. (2021). Adolescents’ digital technology interactions and importance: Associations with demographics and social media frequency. *J. Pediatr.* 236 312–315. 10.1016/j.jpeds.2021.06.005 34119527

[B43] Musetti A. Corsano P. Boursier V. Schimmenti A. (2020). Problematic internet use in lonely adolescents: The mediating role of detachment from parents. *Clin. Neuropsychiatry* 17:3. 10.36131/clinicalnpsych20200101 34908961 PMC8629060

[B44] Nowland R. Necka E. A. Cacioppo J. T. (2018). Loneliness and social internet use: Pathways to reconnection in a digital world? *Perspect. Psychol. Sci.* 13 70–87. 10.1177/1745691617713052 28937910

[B45] Noyan C. O. Enez Darçın A. Nurmedov S. Yilmaz O. Dilbaz N. (2015). Akıllı telefon bağımlılığı ölçeğinin kısa formunun üniversite öğrencilerinde Türkçe geçerlilik ve güvenilirlik çalışması. *Anatolian J. Psychiatry/Anadolu Psikiyatri Dergisi* 16 73–81. Turkish. 10.5455/apd.176101

[B46] Panova T. Carbonell X. (2018). Is smartphone addiction really an addiction? *J. Behav. Add.* 7 252–259. 10.1556/2006.7.2018.49 PMC6174603 29895183

[B47] Rehbein F. King D. L. Staudt A. Hayer T. Rumpf H. J. (2021). Contribution of game genre and structural game characteristics to the risk of problem gaming and gaming disorder: A systematic review. *Curr. Add. Rep.* 8 263–281. 10.1007/s40429-021-00367-7

[B48] Richard F. D. Bond C. F. Stokes-Zoota J. J. (2003). One hundred years of social psychology quantitatively described. *Rev. General Psychol.* 7 331–363. 10.1037/1089-2680.7.4.331

[B49] Sezer M. Cengiz Ö (2025). Erken ergenlik döneminde dijital oyun bağımlılığı, akran ilişkileri ve serbest zaman aktivite düzeyleri arasındaki ilişkinin incelenmesi. *Sportif Bakış: Spor Eğitim Bilimleri Dergisi* 12 62–76. Turkish. 10.70736/spjses.284

[B50] Shah H. A. Househ M. (2023). Understanding loneliness in younger people: Review of the opportunities and challenges for loneliness interventions. *Interact. J. Med. Res.* 12:e45197. 10.2196/45197 37917125 PMC10654910

[B51] Sohn S. Y. Rees P. Wildridge B. Kalk N. J. Carter B. (2019). Prevalence of problematic smartphone usage and associated mental health outcomes amongst children and young people: A systematic review, meta-analysis and GRADE of the evidence. *BMC Psychiatry* 19:356. 10.1186/s12888-019-2350-x 31779637 PMC6883663

[B52] Tokunaga R. S. (2014). A unique problem or the manifestation of a preexisting disorder? The mediating role of problematic Internet use in the relationships between psychosocial problems and functional impairment. *Commun. Res.* 41 531–560. 10.1177/0093650212450910

[B53] Yalçın I Özkurt B. Özmaden M. Yağmur R. (2020). Effect of smartphone addiction on loneliness levels and academic achievement of z generation. *Intern. J. Psychol. Educ. Stud.* 7 208–214. 10.17220/ijpes.2020.01.017

[B54] Yıldız M. A. Duy B. (2014). Adaptation of the short-form of the UCLA loneliness scale (ULS-8) to Turkish for the adolescents. *Dusunen Adam: J. Psychiatry Neurol. Sci.* 27 194–203. 10.5350/DAJPN2014270302

[B55] Zhao C. Ding H. Du M. Yu Y. Chen J. H. Wu A. M. S. et al. (2024). The vicious cycle between loneliness and problematic smartphone use among adolescents: A random intercept cross-lagged panel model. *J. Youth Adolesc.* 53 1428–1440. 10.1007/s10964-024-01974-z 38555341

[B56] Zhou X. Feng B. (2025). Social anxiety and smartphone addiction among college students: The mediating role of loneliness. *Front. Psychiatry* 16:1621900. 10.3389/fpsyt.2025.1621900 40771643 PMC12326073

